# Editorial: When (and How) Is Theory of Mind Useful? Evidence from Life-Span Research

**DOI:** 10.3389/fpsyg.2016.01425

**Published:** 2016-09-22

**Authors:** Francesca Baglio, Antonella Marchetti

**Affiliations:** ^1^IRCCS, Fondazione Don Carlo GnocchiMilan, Italy; ^2^Department of Psychology, Catholic University of the Sacred HeartMilan, Italy

**Keywords:** ToM, typical, clinical, neural, life-span

The Theory of Mind (ToM) paradigm was born almost 40 years ago with the study “Does the chimpanzee have a theory of mind?” by Premack and Woodruff (1978). Several papers (meta-analyses, reviews) have tried to describe the state-of-art, both in general and with respect to specific constructs. From a historical perspective it is possible to identify some relevant turning points in this field of research. ToM, which initially began as a cognitive theory, has subsequently been hybridized to include socio-cultural and/or psychoanalytic perspectives. Within the socio-cultural framework ToM is considered as an outcome of internalization processes in the Vygotskian Zone of Proximal Development; within the psychoanalytic perspective the mentalization process, as ToM is often named, is viewed as a result of the successful relationships with caregivers equipped with a sufficient reflective function. In both cases, intersubjectivity acts as the core concept and the link between ToM and affective relationships have become a new object of investigation. Moreover, there has been increased interest in life-span research from the early stages of life up to the elderly. ToM neural correlates have also been investigated. It has been hypothesized that ToM continues to evolve in a manner that involves both behavioral skills and neural plasticity. Furthermore, the search for novel and more ecological methods aimed to enrich a stronger external relevance. In this regard it has been recognized that “have ToM” and “use ToM” are two deeply different concepts. Furthermore, the use of ToM is linked to the global sphere of social competencies: many studies have tried to understand the predictive role of ToM or at least its connections with other social skills. The present Research Topic (RT) deals with theoretical and empirical contexts with the scope to understand “when” and “how” ToM emerges as a relevant component of social competencies devoted to promoting adaptation and well-being. First, the temporal element, “when,” is referred not only to the chronological dimension of developmental, but also to the subjective relational, circumstances of life, i.e., type of affective relationships, typical vs. non-typical or clinical development and so on. Second, “how” is referred to as the phenomenology of this process, namely the behavioral mode or the neural patterns as well as the connections between them. The RT investigates classic ToM constructs, from the initial concept of “false belief” to pretense and storytelling. Moreover, there are several contributions which aim to use the power of the understanding of subjectivity, in which ToM consists, for educational and therapeutic purposes. The relevance of ToM for the clinical view is supported by the DSM V which includes social competencies in the nosographic framework of developmental impairments (i.e., autism spectrum disorders and social/pragmatic communication disorder) and neurocognitive disorders.

It is possible to cluster the manuscripts that compose the RT around a few principal axes. Some papers offer general considerations about ToM construct. The contribution by Shatz directly addresses the issue raised in by Premack and Woodruff (1978). She argues that the several decades of research concerning ToM have proved that animals and children have a different ToM, since the latter are able to manage both syntaxes and false belief understanding. Battistelli and Farneti, considering meta-representative thinking as equivalent to ToM, identify two domains in which ToM is useful: the reduction of naïve realism and the promotion of metacognitive awareness regarding one's own and other's ignorance (with concrete benefit regarding for example the learning process).

The central concept of belief and false belief has been discussed in many studies as the most debated and utilized ToM measurement. Airenti broached this matter by analysing the difference between the implicit and explicit comprehension of others and the related issue regarding the comparability of verbal and non-verbal tests. Furthermore, Ghrear et al. examine, also from a methodological point of view, the twist between the understanding of false belief and the curse of knowledge. This twist changes along the life-span, and for this reason, new tools are required to evaluate its nature and subsequently the evolution of ToM beyond the “litmus test” of false belief. Finally, Bellagamba et al. investigate the link between the understanding of false belief and the cognitive and affective inhibitory control mechanisms among pre-schoolers. Their findings argue for a specific connection concerning the cold component of inhibitory control. Results are discussed with regard to the link between delayed gratification and mental time travel (i.e., mental projection of themselves into future situations) based on a set of cognitive skills which includes ToM.

Other contributions explore the relation between ToM and some specific abilities deriving from mental representation: pretense, understanding of stories and artistic artifact and the narrative attitude. Dore et al. jointly analyse ToM and social pretend play and subsequently overturn the widely accepted hypothesis of “Play first.” The hypothesis “ToM first” can be supported by naturalistic longitudinal studies. As opposed to the more commonly used training studies, they could investigate both causal directions while monitoring all of the variables potentially involved, with a particular focus on linguistic skills. Through Aesop's Fables, Pelletier and Beatty investigate the bond between the understanding of stories and ToM. Age is positively associated with the ability to infer moral meanings from such stories based on the mental contents of the protagonists rather than on their concrete actions. From an educational perspective, this is important given the role played by the explicit conversation regarding the mental states in the school context. Gilli et al. explore the impact of an interpretative ToM with respect to various issues linked with the representational understanding of the arts. The developmental steps highlight interesting changes in the conceptions about the intentions of both the artists and the art-users, which are progressively included in a more comprehensive view of the respective agency. The understanding of esthetic experience requires critical elements of judgment as well as the recognition of a particular concept of “truth” (artistic artifact vs. fake). Brizio et al. address the social cognition characteristics in adolescence, a period in the life-span which has been scarcely investigated in literature. The classic ToM tasks, suitable for infancy and childhood, are inadequate in this case as they fail to account for the great cultural valence of adolescent social competencies. The latter would be more properly investigated incorporating the narrative tools used by adolescents to cope with challenges and risks of this developmental phase.

Further evidences about the topic are provided by the studies regarding the neural correlates of ToM. Schurz and Perner analyse, on the basis of a previous meta-analysis, nine domain-specific, and domain-general neurocognitive theories and the respective predictions regarding the neural activations while subjects are engaged in six categories of ToM tasks. The failures of the theories in predicting the results are discussed in the context of four critical issues: (1) lack of a clear and shared definition of what is a ToM task, (2) the need for more appropriate knowledge concerning the cognitive processes involved, (3) the need for a more precise anatomical brain mapping, (4) a better understanding of the differences between ToM tasks and Control tasks, which is an emerging topic in this field. Cabinio et al. analyse age-related structural brain changes in relation to a widely used affective ToM task. The negative relation between age and ToM performance is explained by both gray and white matter structural modifications. However, the decline remains within a normal range. The most likely hypothesis is that compensatory mechanisms of brain plasticity aid in maintaining this ToM ability. The study by Marchetti et al. provides both behavioral and neural evidences about Mind Wandering (MW). This study includes the Italian validation of a questionnaire designed to explore the contents of the resting mind. Moreover, the relationship between the ToM contents of the questionnaire and the brain functional connectivity during MW are explored. These evidences show the involvement of areas involved in both affective and cognitive ToM tasks.

The inter-subjective understanding of the subjectivity and the role of attachment process within this dynamic are also addressed in another cluster of papers. Marraffa et al. examine the nativist/modularist theory and the cognitive constructivist theory. They argue that the intersubjectivity promoted by mentalization (named respectively “Mindreading” and “Introspection”) comes from a first-person perspective, connected to mindreading; only at a later point does this scheme tie into a socio-cultural frame, in which the attachment elements are included. Rollo and Sulla analyse through a Vygotskian perspective the influence of maternal mental language on cognitive and socio-cognitive skills in pre-schoolers. Also maternal empathy and child's temperament are proposed as variables that need to be investigated. Rosso et al. examine pre-adolescent and mother dyads in order to characterize the link between the maternal reflective function and pre-adolescents' mentalization process. They observe a lack of connection between the maternal security of attachment and the pre-adolescents' mentalistic abilities, in contrast to the one found with the maternal reflective function. This aspect is especially true regarding negative or ambivalent mental states. These evidences are particularly relevant for focused therapeutic interventions. Similarly, other studies deal with therapeutic and psychoeducational applications. Baimel et al. propose a strategy to understand the other's mind based on behavioral synchrony. The social rituals, which support behavioral synchrony by reducing the psychological distance, can be efficient tools to promote cooperation. The promotion of this mechanism is an implicit way to support ToM as an alternative to overcome the limits of explicit strategies. Bak et al. propose the theoretical basis and application of a Resilience Program. It is an intervention to promote ToM in different contexts for various targets, such as, parents, teachers, and pediatric health care providers. The main characteristic of this program is its flexibility and the possibility to divide it into separate blocks on a case-by-case basis depending on the requirements. Cavallini et al. discuss the results of a ToM training proposed to elderly people. The effectiveness of the training is based on the three fundamental criteria highlighted in the literature for the transfer of acquisitions: (1) repetition, (2) variability of tasks, (3) a learner-oriented approach. From the clinical perspective, Muller and Midgley show the features of the time-limited Mentalization-Based Treatment for Children (MBT-C). It is an adjustment of mentalization-based psychotherapy for borderline patients. Considering mentalization as a set of developing multifaceted abilities allows for a more precise formulation of the various cases. The idea that the development of mentalization is a relational process implies the involvement of parents in different steps of the treatment. This aspect stems from the hypothesis that improved mentalization in parents can strongly produce a similar improvement in that of the child.

Finally, some studies regard ToM in a clinical sample involving participants from school-age to adulthood. Fadda et al. explore the role of ToM in the promotion of moral reasoning in a sample of children with autism spectrum disorder. In these children ToM impairments seem to be connected to a higher proclivity to evaluate actions on the basis of their outcomes. Moreover, they seem more inclined to rigidly comply with moral norms, as in the Piagetian stage of heteronomous morality. Bender et al. investigate the emotive component of ToM with respect to attachment and emotional dysregulation in clinically anxious children. They demonstrate a relationship between the understanding of emotion as mental states and some aspects of anxiety. Ayesa-Arriola et al. analyse whether first episode psychosis patients show a stable ToM impairment over time. In their study, ToM deficits are associated with the neurocognitive status but not with the clinical symptoms. Furthermore, they observe that trait-related mentalizing impairments are found in even in remitted patients.

All contributions presented above elicit some considerations and comments. Developmental researchers appear to remain mostly interested in the quantitative aspect ToM abilities. They study the evolution of these competencies depending on age and specific trainings. Nowadays the qualitative aspect of ToM is more addressed in the clinical field as effects of treatments are also evaluated with respect to the contents of mentalization. In the developmental field the relevance of the ToM quality emerges only in the assessment and training studies where language (i.e., mental language and conversation) is the core of the investigations. Since the beginning of this research paradigm, additional life epochs have been added to both before the original pre-school phase as well as going forward all the way to the elderly one. In the meantime various tasks have been devised to meet these new research requirements as well as to investigate the nascent field concerning ToM's neural correlates. However, there is still a lack of authentic life-span studies performed with cross-sectional methods. On the other hand, longitudinal research is generally performed regarding follow up of clinical and training studies as well as researches on the effects of social relational variables (e.g. security of attachment) on mentalistic abilities in different developmental phases. A relevant future topic of research could concern the long-term developmental patterns of ToM skills in different typical and atypical situations.

## Author contributions

All authors listed, have made substantial, direct and intellectual contribution to the work, and approved it for publication.

### Conflict of interest statement

The authors declare that the research was conducted in the absence of any commercial or financial relationships that could be construed as a potential conflict of interest.
